# The gut microbiome-metabolome dataset collection: a curated resource for integrative meta-analysis

**DOI:** 10.1038/s41522-022-00345-5

**Published:** 2022-10-15

**Authors:** Efrat Muller, Yadid M. Algavi, Elhanan Borenstein

**Affiliations:** 1 Blavatnik School of Computer Science, Tel Aviv University, Tel Aviv, Israel; 2 Sackler Faculty of Medicine, Tel Aviv University, Tel Aviv, Israel; 3 Santa Fe Institute, Santa Fe, NM USA

**Keywords:** Microbiome, Metagenomics

## Abstract

Integrative analysis of microbiome and metabolome data obtained from human fecal samples is a promising avenue for better understanding the interplay between bacteria and metabolites in the human gut, in both health and disease. However, acquiring, processing, and unifying such datasets from multiple sources is a daunting and challenging task. Here we present a publicly available, simple-to-use, curated dataset collection of paired fecal microbiome-metabolome data from multiple cohorts. This data resource allows researchers to easily obtain multiple fully processed and integrated microbiome-metabolome datasets, facilitating the discovery of universal microbe-metabolite links, benchmark various microbiome-metabolome integration tools, and compare newly identified microbe-metabolite findings to other published datasets.

The microbial community residing in the human gut is teeming with metabolic activity and plays a critical role in host physiology and health. The extensive and diverse repertoire of bacterial metabolic functions complements the metabolic capacities of the host, allowing it, for example, to break down otherwise indigestible carbohydrates and to synthesize beneficial vitamins^1^. Microbial metabolites have further been shown to promote gut homeostasis and shape the development and function of the host’s immune system, and may also contribute to gastrointestinal and systemic diseases^2^.

The complete landscape of microbe-metabolite interactions in the gut, however, is still largely unmapped. This gap stems from the limited characterization of bacterial genes, limited scalability of model organism-based (e.g. germ-free mice) or culture-based investigations, the immense portion of yet uncharacterized gut metabolites (the metabolic “dark matter”), and the overall complexity of microbiome-metabolome interactions^3^^,^^4^. Notably, even when restricted to well-characterized taxa and metabolites, the complex gut ecosystem, where host genetics, diet, and other exogenous factors all play a crucial role, renders it difficult to establish robust and confident microbe-metabolite associations^5,6^.

Multiple recent studies have accordingly resorted to joint analyses of microbiome and metabolome data, aiming to systematically evaluate microbe-metabolite links in the human gut^7–10^. These studies have generated paired metagenomic and metabolomic profiles from fecal samples of a cohort of interest, and then applied a variety of statistical tools or advanced computational methods to identify potential associations and patterns in the data. Importantly, however, findings from a single study often do not carry over to other studies or cohorts^11^, and may fail to capture biologically meaningful links^6^. The ability to validate identified microbiome-metabolome associations across multiple cohorts or to pool data from multiple studies to increase statistical power is therefore key to distinguish signal from noise and to demonstrate the generalizability of the obtained findings.

Unfortunately, however, obtaining, processing, and comparing microbiome-metabolome datasets from multiple studies is typically a cumbersome, extremely challenging, and time-consuming process. Initial challenges include downloading the data associated with each study, which are often missing or incomplete, and linking microbiome, metabolome, and metadata sample identifiers in each study. While sharing raw and/or processed metagenomics data is common and relatively standardized in terms of formats and online open-access repositories, metabolomics data is much less standardized and often not being shared in microbiome studies. Once all the raw data have been obtained, they need to be jointly re-processed, which often requires additional expertise or the use of a variety of bioinformatic methods. Making sure taxon and metabolite identifiers can be mapped and compared across datasets is another critical challenge, and may require careful and tedious curation efforts. Schorn et al. have recently addressed some of these challenges by releasing a community resource for linking raw genomic/metagenomic data with metabolomic data^12^, yet, this resource requires proficiency in processing raw data sources and is targeted primarily at identifying and confirming novel links between biosynthetic gene clusters and metabolites.

To address these challenges and to facilitate the reuse of published microbiome-metabolome data for convenient multi-study meta-analysis exploration of microbe-metabolite patterns, we present here a curated dataset collection of paired and processed microbiome-metabolome data from human fecal samples. This resource includes 14 different human gut microbiome-metabolome studies, spanning multiple metagenomic methods, metabolomic methods, cohort demographics, and study designs (Table 1). Researchers can use this resource to easily obtain multiple, curated, and unified microbiome-metabolome datasets in order to compare statistical associations between datasets, benchmark various microbiome-metabolome integration tools, and compare findings from their own dataset to similar datasets – all in much greater convenience and efficiency than before.

**Table 1:** Datasets included in the Curated Gut Microbiome-Metabolome Data Resource.

Dataset name	Ref	Cohort description	No. samples w/ paired data	Longitudinal Y/N	No. HMDB-annotated compounds	No. KEGG-annotated compounds
YACHIDA_CRC_2019	^8^	Patients with colonoscopy findings from normal to stage 4 CRC, and controls	347	No	407	431
FRANZOSA_IBD_2019	^9^	IBD patients and controls (PRISM cohort)	220	No	199	174
SINHA_CRC_2016	^21^	CRC patients and controls	131	No	352	189
HE_INFANTS_MFGM_2019	^14^	Infants on different diets during their 1st year of life	277	Yes	118	111
iHMP_IBDMDB_2019	^15^	HMP2 (iHMP) cohort: Longitudinal samples from IBD patients and controls	389	Yes	455	276
JACOBS_IBD_2016	^16^	IBD patients and their first degree (healthy) relatives	90	No	36	27
POYET_BIO_ML_2019	^20^	Longitudinal samples from healthy BIO-ML (stool bank) donors	164	Yes	255	223
ERAWIJANTARI_GC_2020	^13^	Patients with a history of gastrectomy for GC, and controls	96	No	462	505
KIM_ADENOMAS	^18^	Patients with advanced colorectal adenomas, CRC, and controls	240	No	358	262
MARS_IBS_2020	^19^	Longitudinal samples from patients with IBS and controls	455	Yes	40	36
KANG_AUTISM_2018	^17^	Children with autism and neurotypical children	44	No	58	57
KOSTIC_INFANTS_T1D_2015	^10^	Longitudinal samples from children at risk for T1D (DIABIMMUNE cohort)	103	Yes	138	130
WANDRO_PRETERMS_2018	^22^	Preterm infants during their first 6 months of life. Some developed LOS/NEC	75	Yes	198	199
WANG_ESRD_2020	^23^	Adults with ESRD and controls	287	No	148	87

*CRC* Colorectal cancer, *IBD* Inflammatory bowel disease, *MFGM* Milk fat globule membrane, *BIO-ML* Broad Institute-OpenBiome Microbiome Library, *GC* Gastric cancer, *IBS* Irritable bowel syndrome, *T1D* Type 1 diabetes, *LOS* Late-onset sepsis, *NEC* Necrotizing enterocolitis, *ESRD* End-stage renal disease.

## The curated gut microbiome-metabolome data resource and potential applications

**Fig. 1:**
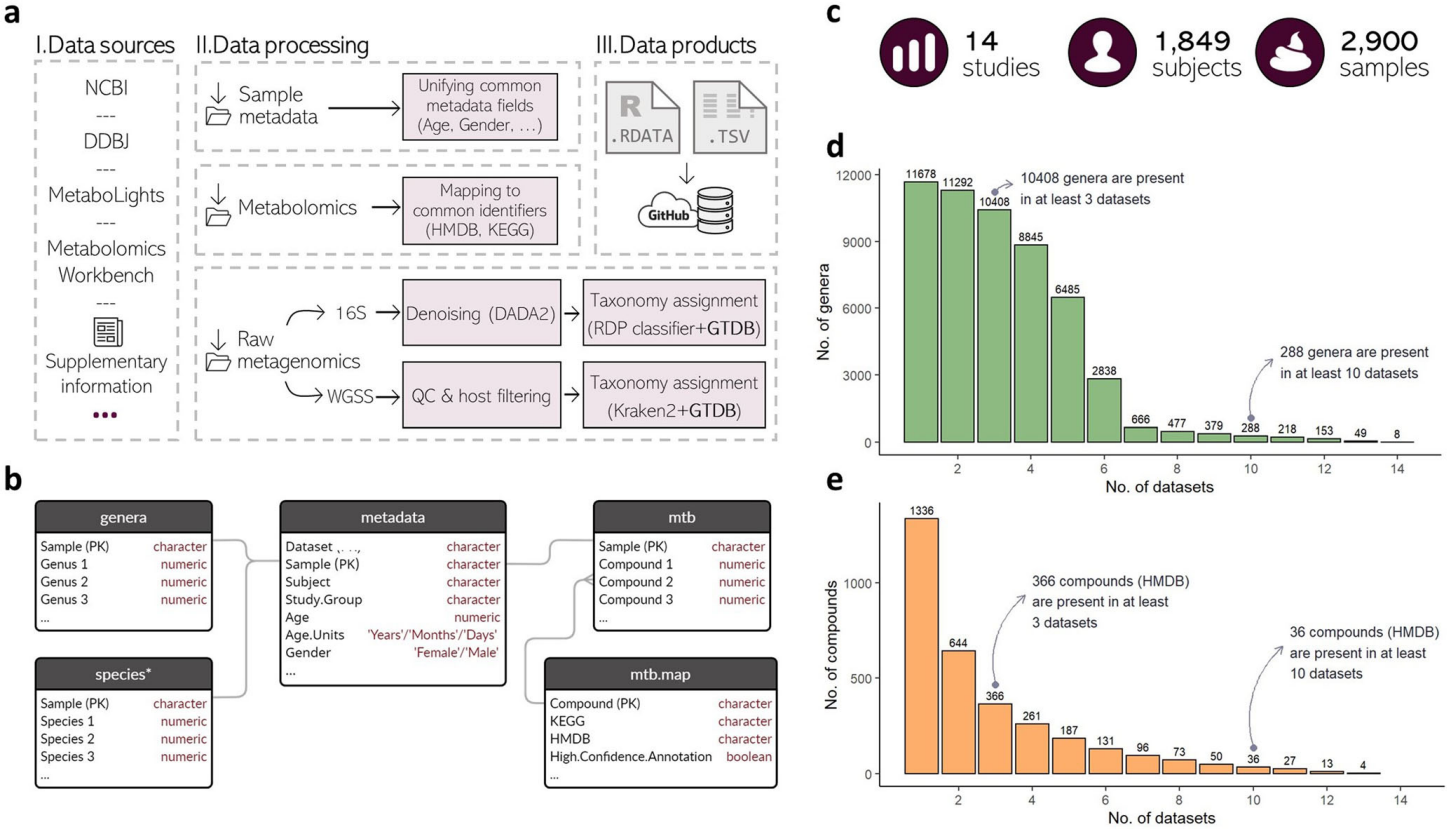
Data resource processing, organization, and statistics. **a** A highlight of data resources and main processing steps of the “curated microbiome-metabolome data resource” (see Methods); **b** A database scheme of the final data products per dataset. Each box describes a specific table and its content and primary key (PK) field. The “species” table is only available for studies with shotgun metagenomic data; **c** Data resource summary statistics; **d** Genera prevalence across datasets. Each bar represents the number of unique genera that appear in at least the specified number of datasets; **e** Metabolite prevalence across datasets, interpretation equivalent to (**d**).

The data resource includes curated and unified data tables from 14 different human gut (feces) microbiome-metabolome published studies from recent years (Table 1, Supplementary Table 1)^8–10,13–23^. Figure 1a highlights the main data sources and key processing steps. For each study we provide 4 processed tables: A genus-level abundance table, a metabolite abundance table, a metabolite identifiers mapping table, and a sample metadata table including sample- and subject-characteristics (Fig. 1b). For studies with shotgun metagenomics we also provided species-level abundance tables. Importantly, microbiome profiles were obtained through processing of raw metagenomics sequencing data, while for metabolite profiles we obtained already processed tables due to the substantial differences between metabolomics instruments and approaches. Where possible, both taxa and metabolite identifiers have been unified, allowing comparison across studies (see Methods). The data for each study are provided both as simple text files (.tsv) and as R-data files (.RData), and are accessible via a public GitHub repository. We further provide detailed documentation and a usage example in a dedicated Wiki page and via script examples also available in the repository. New datasets could be added to the resource by Git pull requests, following the instructions provided in the Wiki section “Adding new datasets”. Overall, 2900 samples from 1849 individuals are currently included in the resource (Fig. 1c). Most of these studies are case-control studies, i.e. they include two study groups, one consisting of individuals with a specific medical condition, and another group of healthy “control” individuals (Table 1).

The described resource, which includes hundreds of unique metabolites and thousands of unique genera that appear in multiple independent datasets (Fig. 1d, e), could be used for different types of meta-analyses or cross-study comparisons involving paired microbiome and metabolome data across health and disease. We specifically identify 3 main categories of analysis use cases, facilitated by this resource: First, this resource can be used for meta-analysis efforts where associations of different types are compared across some or all datasets, aiming to identify robust and consistent signals. Such associations could be identified via a wide range of statistical methods, univariate or multivariate approaches, and using a wide range of features, e.g. taxa at different ranks, microbiome diversity metrics, sample or subject characteristics, metabolite features, etc. Two examples of such meta-analysis efforts are further described below. Second, this resource can be used to benchmark methods related to the joint analysis of microbiome and metabolome data. For example, machine learning methods for predicting metabolite levels based on taxonomic features have been recently proposed but validated on only a very small set of datasets^24,25^. Third, researchers analyzing new microbiome-metabolome datasets can use this resource to add support for findings on their own data, using specific datasets from the resource that resemble their own cohort (studies on the same disease, for example, or using an identical metabolomics method).

Indeed, we recently demonstrated the utility of a similar dataset collection in a large-scale meta-analysis of the relationship between gut microbes and metabolites^26^. In this study we were interested in pinpointing metabolites that are robustly and universally predicted by the microbiota’s composition in a healthy population across multiple studies. Using a combination of random forest regressor models (for predicting metabolites) and random-effects models (for quantifying robustness), we were able to identify 97 metabolites that were robustly well-predicted by the microbiota’s composition. We additionally found that multiple microbiome-metabolite relationships are study-specific, implying that links based on a single study should be interpreted with caution and highlighting the importance of validating findings on additional data sources.

**Fig. 2:**
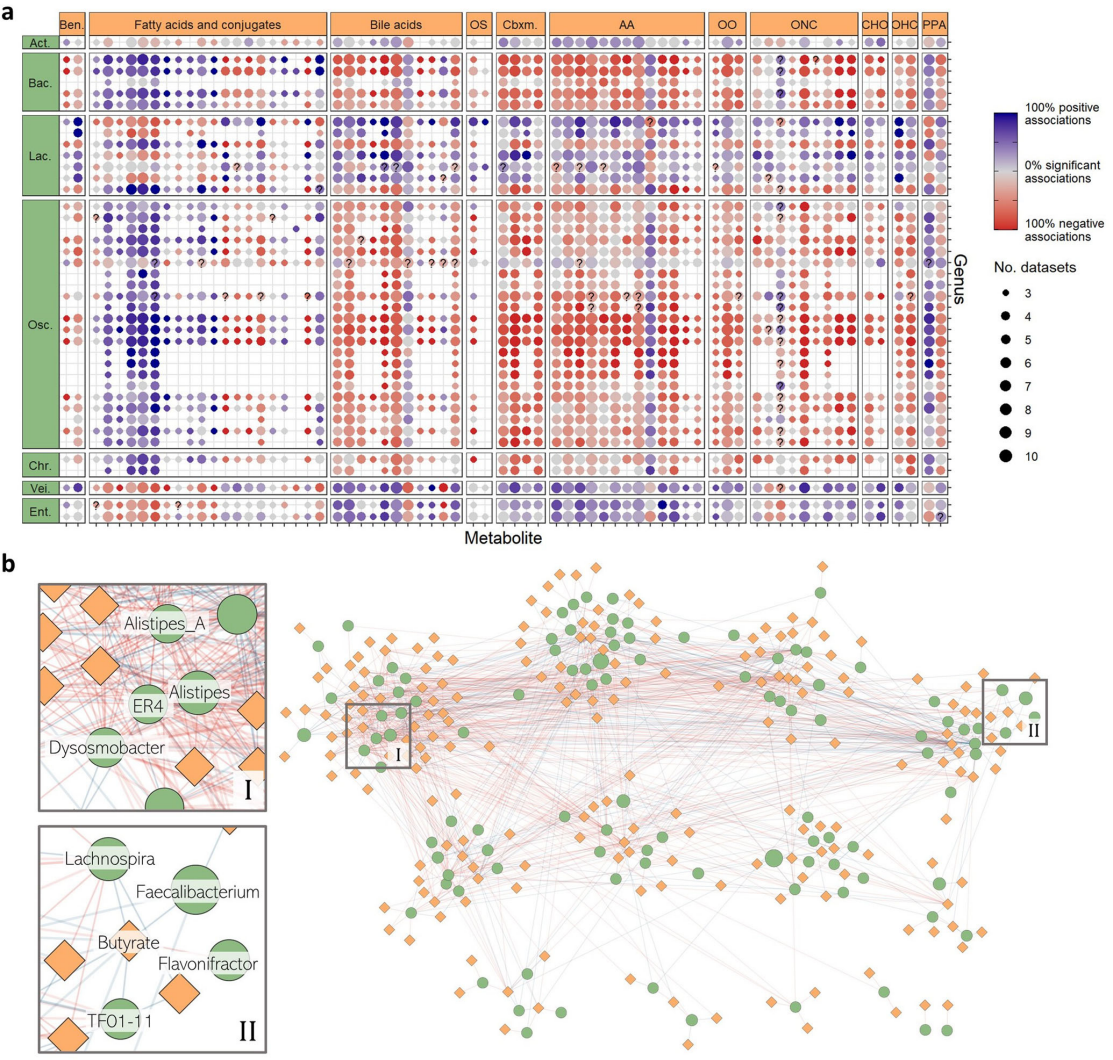
A meta-analysis of genus-metabolite association reveals a dense network of consistent associations. **a** Associations between genera and metabolites were tested using linear models, in each dataset independently and controlling for study groups. The dot plot illustrates association results for the top 70 associated metabolites and the top 40 associated genera. Each dot represents a genus-metabolite pair, dot size represents the number of datasets in which the pair was analyzed, and dot colors represent the percent of datasets in which a significant association (positive or negative) was found (see also Methods). A question mark indicates conflicting results between 2 or more datasets, i.e. at least one significant negative association and at least one significant positive association. Metabolites (grid columns) are grouped by their metabolite classes, abbreviated as follows: Ben. Benzenoids, OS Other steroids, Cbxm. Carboximidic acids, COOH Carboxylic acids and derivatives, AA Amino acids, OO Other organic acids, ONC Organonitrogen compounds, CHO Carbohydrates and carbohydrate conjugates, OHC Organoheterocyclic compounds, PPA Phenylpropanoic acids. Genera (grid rows) are grouped by their *order* taxonomic rank, abbreviated as follows: Actin. Actinomycetales (Actinobacteriota phylum), Bacte. Bacteroidales (Bacteroidota phylum), Lachn. Lachnospirales (Firmicutes_A phylum), Oscil. Oscillospirales (Firmicutes_A phylum), Chris. Christensenellales (Firmicutes_A phylum), Veill. Veillonellales (Firmicutes_C phylum), Enter. Enterobacterales (Proteobacteria phylum), **b** A bipartite network of consistent genus-metabolite associations, identified by a meta-analysis of 11 different microbiome-metabolome datasets from the “curated microbiome-metabolome data resource”. Green nodes represent genera, with node sizes proportional to genus’ average relative abundance, and orange nodes represent metabolites. Edges between genus nodes and metabolite nodes represent a consistent positive (blue) or negative (red) association. Details about the network nodes and edges are available in Supplementary Table 4.

Here, as an additional use-case example, we present another meta-analysis of the microbiome-metabolome relationship, searching for specific genus-metabolite associations that are significant and consistent across multiple datasets (see Methods). For this analysis we included only the 11 non-infant cohorts from our resource, and analyzed a total of 29,708 unique genus-metabolite pairs that appeared in at least 3 different datasets. These pairs included 109 different GTDB genera and 314 metabolites. We used linear models to estimate the association between a specific genus’s abundance and a specific metabolite’s level, while controlling for disease state (i.e. study group). Overall, 132,391 linear models were fitted, of which, 18,075 (13.6%) resulted in a significant genus-metabolite association (i.e. regression coefficient FDR ≤0.05). Comparing the associations’ direction and significance across datasets, we found multiple genus-metabolite pairs associated in some (and often, all) datasets, but interestingly also pairs with conflicting associations in different datasets (Fig. 2a). Notably, genus-metabolite correlations can clearly stem from a direct involvement of the genus in the production, consumption, or degradation of the metabolite, but also from indirect associations related, for example, to interactions between different gut bacteria, or co-abundant metabolites present in specific diets. We similarly emphasize that the analyzed metabolites can be either endogenous to the host, obtained through diet, microbially produced/transformed, or otherwise acquired from the environment. Finding associations across multiple datasets, as facilitated by our resource, potentially increases the likelihood that such associations are microbially driven and represent ubiquitous microbial metabolism, rather than specific host or diet-related associations.

Moreover, to determine which genus-metabolite pairs are consistently associated in a more statistically rigorous manner, we conducted a random-effects meta-analysis using semi-partial correlations derived from the linear regression results (as suggested by Aloe and Becker, 2012^27^). We identified 1101 consistent associations, including in total 104 genera and 195 metabolites (Fig. 2b, Supplementary Table 4; see Methods). Metabolite-associated genera were mostly from the Firmicutes_A phylum but included other phyla as well. Microbe-associated metabolites spanned multiple metabolite classes, with the “organic nitrogen compounds” super-class being enriched for microbially-associated metabolites (odds ratio 3.47 [1.3, ∞], FDR 0.08), and the “organic acids and derivatives” super-class being specifically enriched for Bacteroidota-associated metabolites (odds ratio 3.21 [2, ∞], FDR 0.0004; see Methods).

We additionally examined the bipartite network of consistently associated genera and metabolites, presented in Fig. 2b. A full list of network edges, alongside meta-analysis results, are provided in Supplementary Table 4. We identified several genera with a particularly high number of metabolite associations, including ER4 and Dysosmobacter (both of which were previously identified as Oscillibacter genus), Alistipes, and the recently re-classified Alistipes_A genus (Fig. 2b-I). Even though most of these genera have a relatively low abundance in the human gut (0.36%, 0.66%, 3.3% and 0.1%, respectively, averaged over all samples and datasets in the analysis), they are connected to the highest number of metabolites in the network (51, 44, 43 and 50, respectively). This observation may be explained by at least two potential hypotheses: (i) that these bacteria are highly metabolically active in the gut, and/or (ii) that they possess central ecological roles in the gut microbial ecosystem. The former hypothesis is supported, for example, by a recent study on the newly isolated human commensal *Dysosmobacter welbionis*, where administration of this species to mice was found to strongly influence host metabolism and counteract diet-induced obesity development, with only negligible impact on the overall microbiota composition^28^. Alistipes commensal species are also well-studied for their diverse metabolic functions in the gut^29^. Another recent study, however, supported the latter hypothesis when reporting that based on a gut microbiome analysis of a large Dutch cohort, several Alistipes, Alistipes_A, and unclassified Oscillibacter species were all identified as “keystone species”, predicted to have an important impact on the entire microbiome structure and function^30^. Lastly, we note that analogously to highly-associated genera, there are also a few metabolites that are associated with a high number of genera (over 30). This is perhaps not surprising as some metabolites are imported/exported by dozens of different species^31^, and may in turn be further associated with additional genera by indirect associations.

Another noteworthy highlight from this network is the consistent positive associations between butyrate, a short-chain-fatty-acid with beneficial effects on intestinal homeostasis, and several genera, including Faecalibacterium, Butyrivibrio (formerly classified as TF01–11 genus), Roseburia, Eubacterium_I, Agathobacter, and Lachnospira (Fig. 2b-II; Supplementary Table 4). While the former 5 genera are all known butyrate-producers in the gut^32–34^, Lachnospira does not produce butyrate directly but has an indirect positive effect on other butyrate-producing taxa, upon pectin fermentation^35^. Interestingly, Flavonifractor is consistently *negatively* associated with butyrate in our network, albeit known to be a butyrate-producer^36^. This negative association may reflect an ecological interaction rather than a metabolic one, as Flavonifractor tends to have increased abundance in various host conditions that are also characterized by reduced abundances of major butyrate producers, including disease states, postantibiotic treatments, and during infancy^30,36^.

Future work on consistent genus-metabolite associations (out of the scope of the current study) could include genomic analyses to infer which associations likely stem from known production/consumption capabilities, which association signals are low due to significant species-level variation that masks genus-level findings, which associations “break” in disease states, and whether genera associated with multiple metabolites are also key ecological players in microbial interaction networks.

We note that this resource has several obvious limitations. One major limitation is the substantial difference between various metabolomics platforms and the impact of the used platform on the set of chemical classes that can be detected. Short-chain fatty acids, for example, which are known to be important microbial metabolites in the gut, are mostly detectable by gas chromatography-mass spectrometry and may be therefore missing in datasets using other metabolomics methods^37^. With that in mind, it is important to note that the number of datasets in which a metabolite appears should *not* be used as an indication of its prevalence. Similarly, differences between methods may result in different scales of metabolite values, and hence a direct comparison of metabolite values between studies should be avoided. Lastly, metabolite identification in untargeted metabolomic platforms may vary in its confidence level, which could in turn imply lower confidence of downstream analyses. To allow users of this resource to better address these issues, we provide detailed information about metabolomics methods and identification confidence levels for each dataset in Supplementary Table 3, and specifically mark metabolites with putative identifications (see Methods)^38^. On the microbiome side, differences between 16 S amplicon sequencing and shotgun sequencing, as well as differences in sequencing depth and library preparations, may all effect the resolution and accuracy of the obtained microbiome profiles. We encourage users of this resource to carefully account for these limitations using appropriate analysis approaches (some of which were described above), and to apply caution when interpreting analysis results. Additional recommendations for how to best utilize the resource are available in the Wiki page. Overall, “The Curated Gut Microbiome-Metabolome Data Resource” can facilitate a wide and diverse range of integrated microbiome-metabolome analyses, promote the discovery of robust microbe-metabolite links, and allow researchers to easily place newly identified microbe-metabolite findings in the context of other published datasets.

## Methods

### Data acquisition

We first conducted a literature search to identify human gut microbiome studies where both microbiome and metabolome profiles were obtained from fecal samples. We focused on studies that included at least 40 samples in each study group (or total, in non-case-control studies), for which both metadata, microbiome, and metabolome profiles were available.

Data from each study were either downloaded from public repositories (e.g., SRA, Qiita, Metabolomics Workbench), obtained from studies’ supplementary information, or shared directly by the corresponding authors. For microbiome data we obtained raw fastq files, from either 16 S rRNA gene sequencing or whole genome shotgun sequencing (WGSS), or used processed tables if raw data was unavailable (Supplementary Table 1). For metabolome data, both “targeted” and “untargeted” metabolomic approaches were considered. Untargeted metabolomics are methods for comprehensively analyzing all measurable analytes in a sample, most of which are typically unknown molecules, while targeted metabolomics are methods that measure a predefined set of chemically characterized and annotated metabolites. Untargeted datasets were only included if at least a substantial portion of metabolites were identified by name, KEGG ID^39^, or HMDB ID^40^. Importantly, we obtained only metabolome data already processed and quality-controlled by the authors of the original publications, typically provided as text files or excel tables, and with metabolite identifications made as part of the original publications as well (Supplementary Table 3).

Additional details about the original data obtained per study can be found in Supplementary Table 1. All studies whose data were included in this collection were complied with the relevant ethical regulations and reported the specific details in the original publications^8–10,13–23^.

### Processing and unification

Microbiome taxonomic profiles were obtained by either re-processing raw 16 S rRNA gene sequencing data using QIIME2 (version 2019-1)^41^ and DADA2^42^, or re-processing raw WGSS using fastp^43^ for quality control, bowtie2^44^ for host read filtering, and kraken2-braken^45,46^ for taxonomy assignments. For both data types and processing pipelines, we used the Genome Taxonomy Database^47^ (GTDB) as the reference database for taxonomy assignments, as it is specifically designed to provide consistent and comprehensive taxonomy for bacterial genomes. To further assure comparable taxonomic profiles, we also collapsed taxonomy abundance tables into the genus level (species-level tables are available as well for WGSS datasets). Finally, values were converted to relative abundances, i.e. taxa abundances sum to 1 for each sample.

For metabolomics data, we left the original metabolite features unchanged, but added a mapping file from the original feature names to common metabolite identifiers, namely KEGG ID’s and HMDB ID’s, where possible (Fig. 1b). These were either available in the originally published datasets, or obtained using MetaboAnalyst’s compound ID conversion utility^48^. Table 1 lists the number of HMDB/KEGG annotated metabolites per dataset. Importantly, metabolite annotations in untargeted metabolomics may vary in their level of confidence^49^. We therefore mentioned metabolite annotation methods per dataset, as reported by the authors of the original publications, in Supplementary Table 3, and additionally marked specific metabolites as “High.Confidence.Annotation=FALSE” (“mtb.map” tables, Fig. 1b) in cases where users should treat the provided annotation with caution (see Wiki for further details). We finally assured consistent *sample* names across microbiome profiles, metabolome profiles and sample metadata. Additional processing details can be found in our Wiki page (https://github.com/borenstein-lab/microbiome-metabolome-curated-data/wiki/The-Curated-Gut-Microbiome-Metabolome-Data-Resource) and in Supplementary Tables 1–3.

### Data structure and file types

Overall, we provide 4 processed tables for each study: A genus-level relative abundance table, a metabolite abundance table, a sample metadata table and a metabolite identifiers mapping table. In the former three tables, each row represents a sample (sample names are given in the first column) and each column represents a feature (either genus abundance, metabolite levels, or any sample- or subject-characteristic provided in the available metadata). The metabolite identifiers mapping table describes mappings from original metabolite identifiers (as in originally published data) to KEGG or HMDB identifiers. Species-level abundance tables are provided as well for studies that used WGSS. Figure 1b illustrates the final data scheme per study.

Tables were saved as both tab-delimited text files (.tsv) and as R-data files (.RData), and are downloadable via a public GitHub repository (https://github.com/borenstein-lab/microbiome-metabolome-curated-data).

### Genus-metabolite associations meta-analysis

For this analysis, we included only the 11 non-infant cohorts from our resource, and allowed more than one sample per individual if present. After removing rare genera (defined here as <25% non-zero values or average abundance <0.1%, averaged over all datasets in the analysis), and taking only HMDB-annotated metabolites, we extracted a list of genus-metabolite pairs that appeared in at least 3 datasets. For each such pair we fitted a linear model using the following formulation:

$$Metabolite\sim (Intercept) + Genus + Study\_Group$$

We applied a log-transformation (with pseudo count 1) to metabolomic data and an arcsine square root transformation to genera relative abundances before fitting the regressors, as often applied to such data before linear modelling^19^. The *StudyGroup* covariate was omitted in studies with no defined study groups. Per linear model, we report the adjusted R square, the coefficient of the *Genus* variable, it’s associated p-value, and for the subsequent meta-analysis we also report the semi-partial genus-metabolite correlation^27^. FDR was used to control for multiple hypothesis testing per dataset.

To synthesize results across studies we used random-effects models (REM) per genus-metabolite pairs using the semi-partial correlation as the effect size. The ‘metacor’ function from R ‘meta’ package was used for fitting REM’s, with the HAKN correction enabled and with otherwise default settings^50^. Pairs were finally defined as *consistently associated* if the REM’s FDR-corrected p value was below 0.1, and the direction of association was determined by the sign of the REM’s pooled effect size. Supplementary Table 4 includes additional statistics recorded per REM.

We analyzed whether some metabolite super-classes, as labelled in HMDB, are enriched with microbe-associated metabolites using a Fisher’s exact test. We applied this enrichment test once for all microbe-associated metabolites and once for each phylum separately, and FDR-corrected all Fisher tests p values. Finally, we used CytoScape to visualize the network of consistent associations, with the “GLay community clustering” plugin for network layout^51,52^.

## Supplementary information

Supplementary Tables

## Data Availability

The dataset collection is available at https://github.com/borenstein-lab/microbiome-metabolome-curated-data. Documentation is available at the repository’s Wiki site at: https://github.com/borenstein-lab/microbiome-metabolome-curated-data/wiki/The-Curated-Gut-Microbiome-Metabolome-Data-Resource. To obtain the original data as provided by the original publications, see details in Supplementary Table 1.

## Code availability

Main data processing scripts, and the R notebook containing the meta-analysis described herein, are available at https://github.com/borenstein-lab/microbiome-metabolome-curated-data.
